# A Review on the Electroless Deposition of Functional Materials in Ionic Liquids for Batteries and Catalysis

**DOI:** 10.3389/fchem.2019.00085

**Published:** 2019-02-20

**Authors:** Abhishek Lahiri, Giridhar Pulletikurthi, Frank Endres

**Affiliations:** Institute of Electrochemistry, Clausthal University of Technology, Clausthal-Zellerfeld, Germany

**Keywords:** ionic liquids, electroless deposition, catalysis, batteries, galvanic displacement

## Abstract

Developing functional materials via electroless deposition, without the need of external energy is a fascinating concept. Electroless deposition can be subcategorized into galvanic displacement reaction, disproportionation reaction, and deposition in presence of reducing agents. Galvanic displacement reaction is a spontaneous reduction process wherein the redox potentials of the metal/metal ion in the electrolyte govern the thermodynamic feasibility of the process. In aqueous solutions, the galvanic displacement reaction takes place according to the redox potentials of the standard electrochemical series. In comparison, in the case of ionic liquids, galvanic displacement reaction can be triggered by forming metal ion complexes with the anions of the ionic liquids. Therefore, the redox potentials in ILs can be different to those of metal complexes in aqueous solutions. In this review, we highlight the progress in the electroless deposition of metals and semiconductors nanostructures, from ionic liquids and their application toward lithium/sodium batteries, and in catalysis.

## Introduction

**Figure 1:**
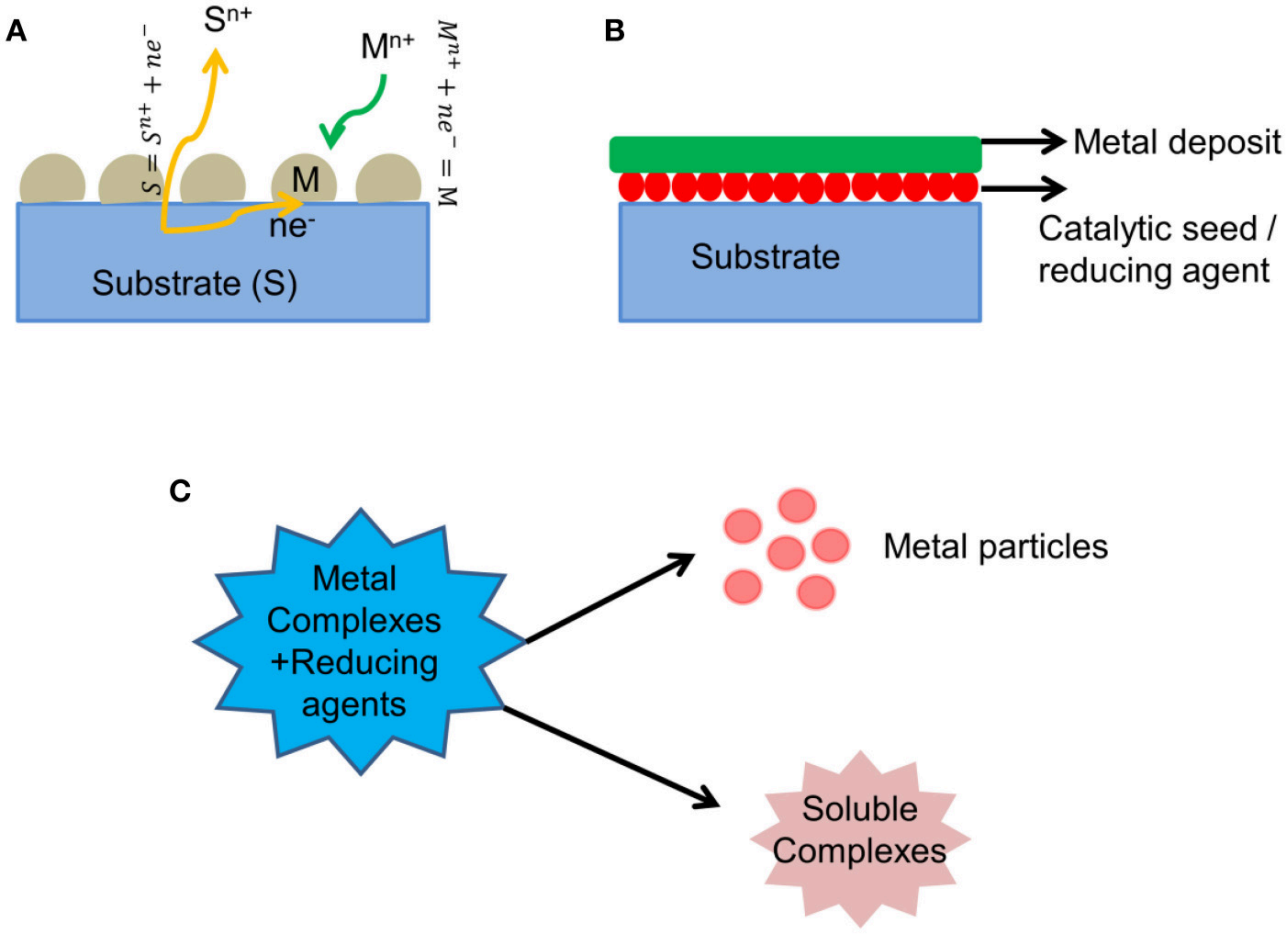
Schematic diagram of various electroless deposition processes **(A)** Galvanic displacement reaction **(B)** Reduction using reducing agents **(C)** Disproportionation reaction.

A simple route for developing various metal and semiconductor nanostructures is one of the major challenges in materials research, which has a significant impact in the fields of energy storage and conversion, catalysis, sensors, photonics and optoelectronics, and biology. Electroless deposition is an elegant and versatile technique for metal plating as well as for developing various metal nanostructures. Electroless deposition can be subdivided into three categories: (1) Galvanic Displacement reaction: Figure 1A shows a schematic diagram of this process. In this process, when exposing a less noble metal containing solution (S) to a more noble metal (M), a spontaneous electrochemical reaction (redox reaction) takes place wherein the more noble metal is reduced by the Electrons generated from the less noble metal, as shown in Equations (1, 2). This technique is used to develop various nanostructures which can be used for different applications in batteries and catalysis.

(1) $$\begin{array}{l} S=S^{n+}+ne^{-} \end{array}$$

(2) $$\begin{array}{l} M^{n+}+ne^{-}=\text{M} \end{array}$$

(2) Using a reducing agent: In this process, the employed reducing agent undergoes oxidation and provides electrons to the metal ions present in the electrolyte. When exposing the substrate to a metal ion containing electrolyte, spontaneous metal deposition occurs on the substrate as shown in the schematic diagram in Figure 1B. This technique is generally used for developing metal coatings over insulating or non-conductive substrates.

(3) Disproportionation reaction: In this process, the metal complex in presence of certain substances such as acids/salts or carbon monoxide, leads to the formation of metal particles, and other soluble products as shown in the schematic diagram in Figure 1C. This technique is usually used to generate metal nanoparticles which can be used as catalysts.

In aqueous systems, the galvanic displacement reaction occurs according to the standard electrochemical series wherein the redox reactions mainly depend on the concentration of metal ions, pH, and temperature. Furthermore, the electroless deposition is usually limited to a few monolayers without the addition of a catalyst. A change in the metal ion speciation in aqueous solutions might also result in a deviation from the standard electrochemical potentials, which has not been studied in great detail (Abbott et al., 2011, 2018), but might open up a few new opportunities for developing functional materials.

In direct comparison to aqueous solutions, ionic liquids possess advantages for favoring the galvanic displacement reaction. Ionic liquids are entirely made up of cations and anions, and can be divided into three subcategories of protic, aprotic, and deep eutectic solvents (DES) (Smith et al., 2014; Greaves and Drummond, 2015; Watanabe et al., 2017). In the last couple of years, the addition of water and molecular solvents in ionic liquids have also been explored as electrolytes for different applications and been described as the “fourth evolution in ionic liquids” (MacFarlane et al., 2018). This makes a large number of ionic liquids and its mixtures as possible electrolytes to tune the redox reactions (M/M^n+^) and to trigger the galvanic displacement process. However, compared to aqueous electrolytes, there is no universal electrochemical series in the case of ionic liquids due to the lack of a standard reference electrode potential.

As only ions are present in ionic liquids, by changing the anions of the ionic liquids, the speciation of metals/semiconductor ions can be altered (Borisenko et al., 2018; Lahiri et al., 2018a). A change in the speciation leads to a different electrochemical behavior and has also shown to affect the morphology of the deposit (Pulletikurthi et al., 2013, 2014; Borisenko et al., 2018; Lahiri et al., 2018a). Interestingly, it was reported that changing the cations of the ionic liquids also affects the morphology of the electrodeposits (Zein El Abedin et al., 2006; Al-Salman and Endres, 2009; Ispas et al., 2010). The changes in the morphology of the electrodeposit were related to the difference in the interfacial structure of the ionic liquid at the electrode/electrolyte interface (EEI) (Endres et al., 2010). Unlike aqueous systems which form a double layer structure at the EEI, ionic liquids form a multilayered structure and the addition of metal ions affects this EEI structure (Lahiri et al., 2015a; Carstens et al., 2016), which influences the deposition kinetics. Therefore, in ionic liquids, a variation in the combination of both anions and cations can change the electrochemical behavior of metal/semiconductor deposition, which would also be the case for electroless deposition. In batteries, synthesis of various nanostructures with a high surface area and mechanical stability is important in order to develop a long-term stable battery. Electroless deposition in ionic liquids shows a promising route to obtain such nanostructures. Furthermore, in catalysis, not only is a high surface area important, but a high number of catalytic activation sites are also necessary. Such control over morphology and tunable active sites can be achieved in ionic liquids which will be shown in the review.

Thus, based on the above brief description, it is clear that electroless deposition in ionic liquids differs from aqueous-based electrolytes. Numerous studies of electroless deposition in aqueous systems can be found in literature, yet little has been reported from ionic liquids. In this short review, we will present results on electroless deposition of metals in ionic liquids and show the application of the obtained nanostructures for batteries and catalysis. Finally, we will propose the various challenges and future prospects of electroless deposition in ionic liquids.

## Electroless Deposition of Noble Metals

The first example of galvanic displacement reaction of silver (Ag) on Cu was shown by Abbott et al. (2007) in a DES composed of choline chloride (ChCl) and ethylene glycol (EG). Electroless deposition of Ag is an important industrial process for printed circuit boards (PCBs) to prevent degradation of the copper surface and is usually done by plating silver on copper from an AgNO_3_/HNO_3_ solution. Compared to the corrosive nature of the aqueous solution which also affects the copper in PCBs, an ionic liquid or DES does provide a safer alternative. Secondly, in aqueous solutions for the electroless deposition to prolong beyond few nanometers, a palladium catalyst is used (Shipley, 1961; Djokic, 2002). However, in the case of ionic liquids, it was shown that the galvanic displacement reaction of silver continued beyond a few nanometers without the use of any additional catalyst. In ChCl:EG eutectic ionic liquid, the galvanic displacement reaction took place according to Equation (3).

(3) $$\begin{array}{l} Ag_{IL}^{+}+Cu\left(s\right)=Cu_{IL}^{+}+Ag\left(s\right) \end{array}$$

**Figure 2:**
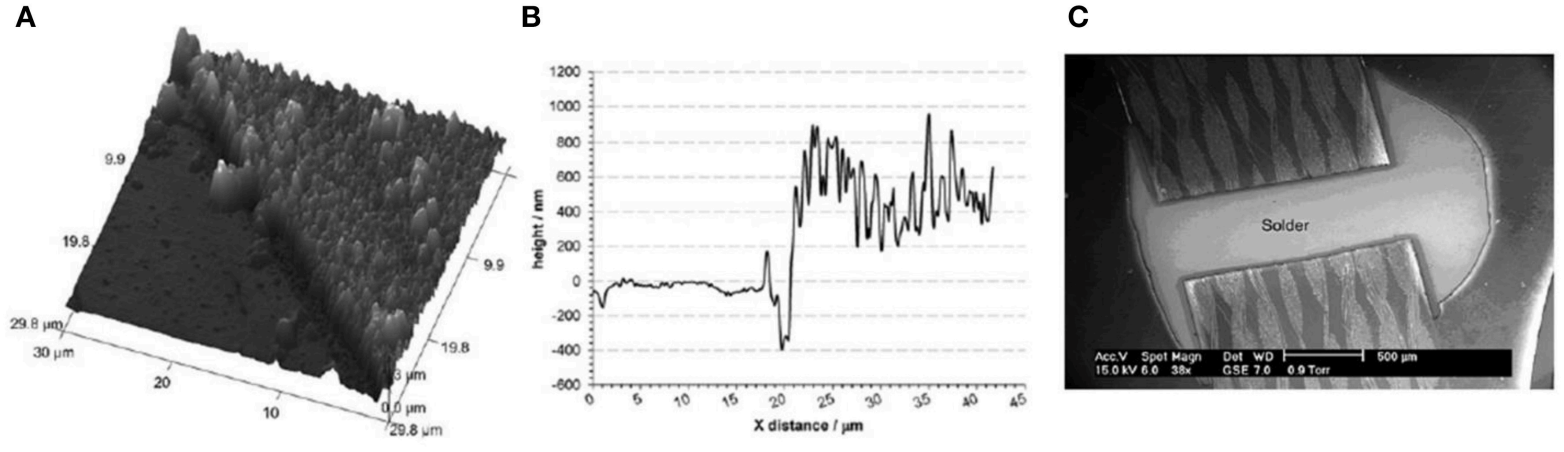
**(A)** 3D AFM image of electroless deposited Ag on Cu **(B)** AFM height profile of the electroless deposited Ag on Cu shown in Figure 1A **(C)** Ag deposited on a printed circuit board. Reproduced from Abbott et al. (2007) and Abbott et al. (2008) with permission from The Royal Society of Chemistry and Elsevier.

Figures 2A,B shows the AFM image of the deposit along with the height profile of Ag deposition on Cu, respectively. It is evident from Figure 2A that Ag electroless deposition on Cu is thick and from the height image in Figure 2B, the thickness was found to be ~500 nm. This shows that electroless deposition of Ag on Cu from ionic liquids is clearly different from aqueous electrolytes where thermodynamics governs the redox potential and the reaction would stop in aqueous electrolytes once the Cu is covered with Ag. However, this was not the case for ILs as reported in Abbott et al. (2007). The authors reported from quartz crystal microbalance (QCM) investigations that during a controlled electroless deposition wherein copper was first electroplated on Au, with time, all the copper was replaced by Ag. Based on the results of scanning electron microscopy (SEM), Atomic Force Microscopy (AFM), and QCM, it was concluded that the electroless deposition of Ag on Cu might be limited by the diffusion of Cu^+^ ions. Temperature and different silver salts were also found to influence the deposition of Ag.

Different silver salts (AgNO_3_, Ag_2_SO_4_, Ag(acetate), and AgCl) were investigated for silver plating. The best and most consistent result for electroless deposition of Ag on Cu was obtained from AgCl in the DES (Abbott et al., 2008). This means that the anion of the Ag salt alters the electroless deposition process which might be due to the change in speciation. However, Ag speciation studies are still lacking in DES which would have provided more information regarding the deposition quality as well as reaction kinetics. From the above studies, it appears that Cl^−^ ions can trigger the galvanic displacement reaction at the electrode/electrolyte interface. With a change in temperature from 20 to 45°C, the deposition rate was found to increase from 45 to 110 nm min^−1^ (Smith et al., 2010). Based on the results of the galvanic displacement of Ag on Cu, the authors also performed a pilot scale study for Ag plating on Cu in PCBs (Smith et al., 2010). For an industrial process, the rate of deposition must be increased to 0.2 μm min^−1^ which was achieved by increasing the Ag concentration in the liquid. Figure 2C shows the image of Ag coated Cu in a PCB using electroless deposition in the liquid. This clearly shows that it is possible to scale-up electrochemical processes in ILs and deep eutectic solvents with comparable/better results than the existing methods without the use of sacrificial precious metals.

Silver was also plated on Cu from a 1-butyl-3-methylimidazolium bromide (BMImBr)-AgBr electrolyte (Grishina and Ramenskaya, 2017). It was shown that the displacement reaction leads to the formation of CuBr_2_ which dissolves in the electrolyte turning the solution to pale violet. From such a liquid, a silver coating thickness of up to 10 μm could be achieved. By studying the reaction kinetics, the authors showed that the galvanic displacement process took place in two stages. The rate constant in the first stage was found to be 5.1 × 10^−5^ s^−1^ which increased to 5.3 × 10^−4^ s^−1^ in the second stage and was related to the decrease in charge transfer resistance (Grishina and Ramenskaya, 2017). Although, compared to DES, a much thicker Ag layer could be achieved, the viscosity of BMImBr is higher than DES and therefore the removal of ionic liquids after the displacement process might be an issue. Furthermore, questions regarding the reuse of the BMImBr-AgBr electrolyte for plating silver on copper for practical applications remain unanswered.

Wang et al. studied the use of aqueous based choline chloride to study the galvanic displacement reaction of Ag on Cu with different silver salts of AgNO_3_, AgCl, and Ag_2_SO_4_ (Wang et al., 2013a). It was shown that the mole ratio of ChCl and H_2_O affected the deposition of Ag from AgNO_3_. Furthermore, as observed previously by Abbott et al. with increase in temperature, the rate of deposition was shown to increase and by determining the deposition rate at various temperatures, the activation energy for Ag deposition was found to be 13 kJ mol^−1^. However, from XRD analysis, the authors observed both Ag and Cu peaks which suggests that the thickness of Ag deposited was very low. Furthermore, the lack of adhesion tests along with limited solubility of AgCl and Ag_2_SO_4_ in the electrolyte questions the applicability of aqueous based electrolytes for metal plating.

The same authors also studied the electroless deposition of Pd on Ni-P coated Cu sheets using ChCl-H_2_O electrolyte containing PdCl_2_ (Wang et al., 2015a). Pd is a versatile catalyst to promote many organic reactions (Miyaura and Suzuki, 1995) as well as for hydrogen evolution reactions (Pentland et al., 1957) and therefore the electroless deposition becomes a simple technique to develop Pd nanostructures for catalysis applications. For electroless deposition of Pd on Ni-P coated Cu, plating parameters such as ChCl concentration, PdCl_2_ concentration, pH, and temperature of the electrolyte were investigated. The pH of mixtures of ionic liquids and water can in principle be measured e.g., ChCl-H_2_O mixtures. However, the pH of pure/neat aprotic ionic liquids cannot be defined. The galvanic displacement reaction involved the oxidation of nickel and the reduction of Pd^2+^ ions. The authors showed that as the concentration of ChCl in the electrolyte increased from 20 to 120 g L^−1^, the deposition rate of Pd decreased from 2 to 0.6 μm h^−1^ which was attributed to the change in the coordination of Pd^2+^ ions in ChCl. Unfortunately, the speciation of Pd ions in the electrolyte was not analyzed by the authors. The microstructure of the deposit was also found to change with different immersion times. With deposition time of < 5 min, spherical structures were obtained whereas with increase in time to 30 min, nanosheet like structures were observed. Increasing the electrolyte temperature from 55 to 75°C at a fixed ChCl:PdCl_2_ ratio led to an increase in deposition rate from 1.1 to 2 μm h^−1^. The change in pH of the electrolyte from 0.6 to 2 did not affect the deposition rate significantly. At an optimum electrolyte composition of 40 g L^−1^ ChCl, 2 g L^−1^ PdCl_2_, and at a temperature of 60°C, Pd deposition of about 3 μm in thickness could be achieved in 90 min. Finally, the authors tested the corrosion resistance of Pd coated Ni-P/Cu in 3.5% NaCl and showed that Pd indeed improves the corrosion resistance of the material. Although, water based ionic liquids showed the possibility of galvanic displacement reaction, the applicability of the deposited Pd as catalyst was not investigated. Furthermore, the microstructure was shown to change from spherical to nanosheet like structure with deposition time. Such changes in microstructure are particularly interesting in catalytic applications. The lack of such studies makes it hard to evaluate the applicability of electroless deposition processes compared to regular electrodeposition processes.

Electroless deposition of gold was investigated from a few ionic liquids (Aldous et al., 2007; Ballantyne et al., 2015; Wang et al., 2015b). Gold deposition from chloroauric acid (HAuCl_4_) in ChCl-H_2_O was investigated on Ni-P coated Cu (Wang et al., 2015b). The authors showed that with an increase in HAuCl_4_ concentration in the electrolyte (from 0.5 to 2 g L^−1^), the rate of deposition increased from 5 to 22.5 nm min^−1^. However, with higher concentration (2 g L^−1^), the gold coating peeled off from the substrate. The change in ChCl concentration also affected the deposition rate. With decrease in ChCl concentration again the coated gold did not adhere to the surface whereas with increase in ChCl concentration, the gold deposition rate decreased. The change in the deposition rate with ChCl concentration seems to be due to a change in the coordination of HAuCl_4_ with ChCl. However, the lack of the information regarding Au^3+^ coordination in the electrolyte makes it difficult to get a clear picture about the deposition process. The plating temperature also affected the rate of deposition. At 60°C, gold deposited at 6 nm min^−1^, which increased to 13 nm min^−1^ at 90°C. The change in pH of the electrolyte from 0.5 to 3 did not influence the gold deposition rate. For a practical application, the authors also investigated the lifetime of the electrolyte by measuring the absorption spectra of the Au^3+^ ion as a function of time. They found that the solution was relatively stable over time and a loss in absorption of only ~5% was observed after about 2 months. Based on this work it is clear that galvanic displacement of gold is possible from water based ionic liquids. However, the non-adhesive nature of the film rises concerns about the applicability of this electrolyte. It appears that slow rate of deposition is better for obtaining a stable gold coating. Further studies are required e.g., by preparing suitable electrode materials by surface modification to achieve good Au adhesion from the water based ionic liquid electrolyte.

A comprehensive study regarding metal-ion speciation of Au^+^ and how it influences the deposition rate and morphology was performed by Ballantyne et al. The Au^+^ speciation was studied for three different gold salts [AuCl, AuCN, and KAu(CN)_2_] in a deep eutectic solvent composed of ChCl and 1, 2-ethanediol (Ballantyne et al., 2015). Prior to galvanic displacement reactions, the authors tested the gold electrodeposition from the three salts on Pt. The cyclic voltammetry (CV) experiments revealed that a complete reversible process for the gold deposition and stripping occurred for AuCl whereas for both AuCN and KAu(CN)_2_ no noticeable deposition peaks were observed.

**Figure 3:**
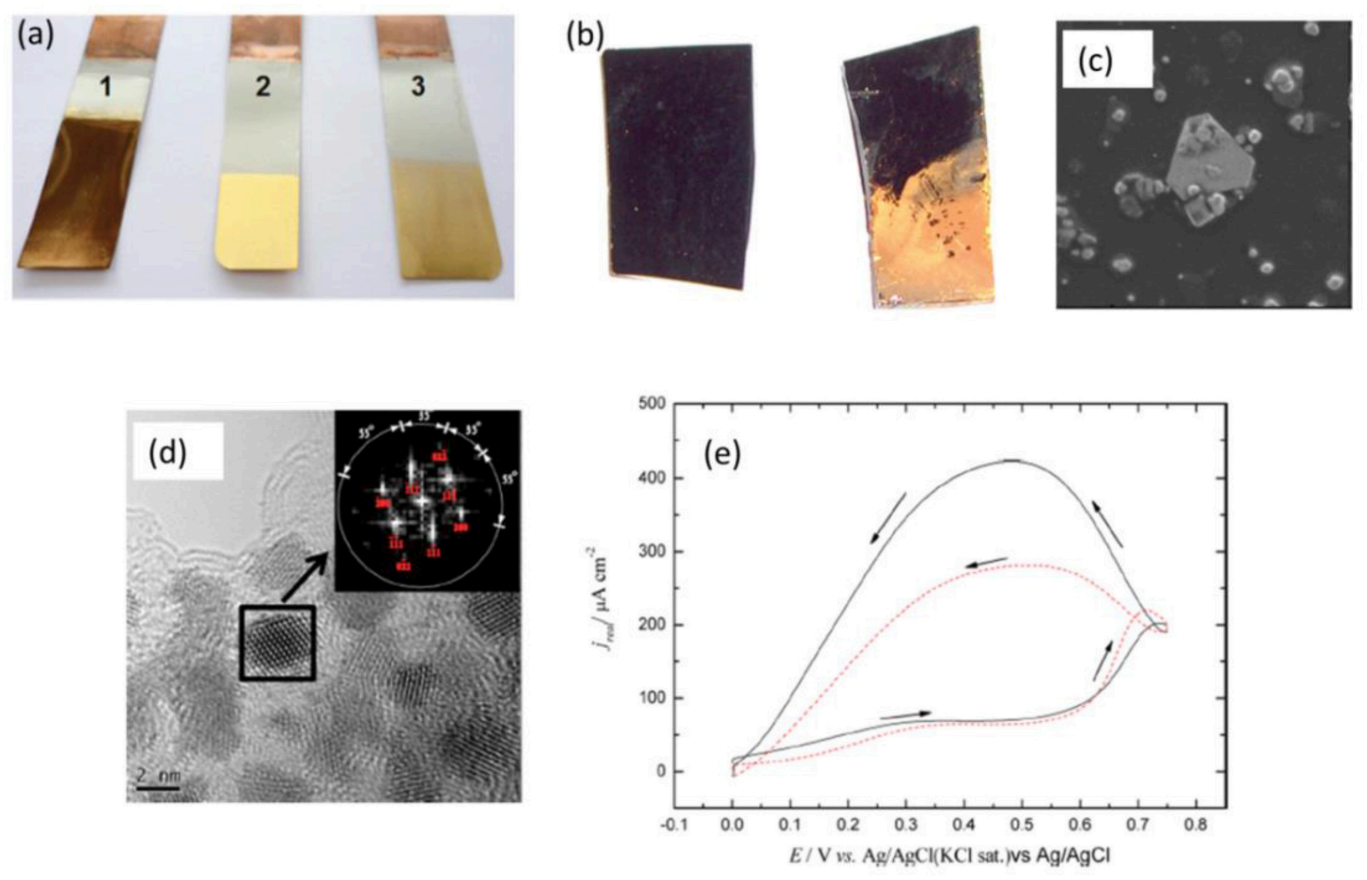
**(a)** Electroless deposition of Au on nickel from (a1) AuCl (a2) AuCN (a3) KAu(CN)_2_ in ChCl-1, 2-ethanediol **(b)** Electroless deposition of Au from H[AuCl_4_] in EMImTFSA in presence of HTFSA on glassy carbon **(c)** Gold nanoplates formed by electroless deposition on glassy carbon from H[AuCl_4_] in EMImTFSA in presence of HTFSA **(d)** Electroless deposition of Pt nanoparticles **(e)** Methanol electrooxidation on Pt nanoparticles formed by electroless deposition. Reproduced from Aldous et al. (2007), Zhang et al. (2013) and Ballantyne et al. (2015) with permission from The Royal Society of Chemistry and American Chemical Society, Copyright 2007, 2013 American Chemical Society.

This clearly indicated that Au speciation in the ionic liquid was different. From extended X-ray absorption fine structure (EXAFS) measurements, the authors showed that for AuCl, a coordination number of two was obtained leading to the formation of [AuCl_2_]^−^ species in the ionic liquid. However, in presence of AuCN and KAu(CN)_2_, a mixed coordination of [AuCl(CN)]^−^ was observed and no exchange of ligand between CN and Cl took place. Therefore, from the CV, no clear deposition and stripping peaks were obtained for the Au salts in liquids containing cyanide ions. On performing electroless deposition of Au onto nickel from the three electrolytes, the plating rate was found to be different. From AuCl, a plating rate of 1.16 nm min^−1^ was obtained whereas from AuCN and KAu(CN)_2_ the deposition rate decreased to 0.76 and 0.37 nm min^−1^, respectively. Figure 3a shows the optical image of Au plated on nickel from three different Au salts. Although, a thick gold film was obtained from AuCl (showing a dark brown color in Figure 3a, 1), the Au deposit did not adhere strongly to the nickel substrate, and could be easily peeled off. The best gold finish on nickel with a good adhesion property was obtained from the ionic liquid containing AuCN (Figure 3a, 2) whereas the deposition from KAu(CN)_2_ did not give a good plating quality (Figure 3a, 3). Thus, this study clearly revealed that metal ion speciation is not only an important factor to control the deposition rate but also to develop better coating materials. Further work based on these studies can be performed to obtain different gold morphologies to be applied in catalysis.

A unique way of proton induced electroless deposition of Au was shown by Aldous et al. (2007) from the ionic liquid 1-butyl-1-methylimidazolium bis(trifluoromethylsulfonyl)amide ([BMIM]TFSA). The authors showed that gold could be deposited on glassy carbon (Figure 3b) without applying any potential from H[AuCl_4_] in [BMIM]TFSA. They argued that [AuCl_4_]^−^ gets adsorbed on specific sites of the glassy carbon electrode and the H^+^ assists in the reduction of gold ions via a disproportionation reaction. It was further shown that addition of HTFSA to the electrolyte facilitated the electroless deposition process. Interestingly, the electroless deposition led to formation of triangular facet gold nanoplates as well as gold nanoparticles as shown in Figure 3c. However, changing the anion of IL from TFSA^−^ to [PF_6_]^−^ or [BF_4_]^−^ did not lead to gold deposition. Although an interesting result of developing tunable gold nanoplates was demonstrated, no further studies were taken up to identify the parameters for producing only triangular shaped gold nanoplates. Gold nanoparticles and nanoplates have immense applications in biosensing (Zeng et al., 2011), plasmonic (Anderson et al., 2013), catalysis (Thompson, 2007), and so forth. Therefore, exploiting electroless deposition to directly obtain tunable plasmonic properties opens new avenues for material synthesis. Furthermore, speciation of Au might have played a key role to promote and develop different gold nanostructures which needs further investigation.

Gold deposition from H[AuCl_4_] in 1-butyl-1-methyl-pyrrolidinium dicyanamide ([Py_1, 4_]DCA) was studied on copper at different temperatures of 20–80°C (De Sá et al., 2013). The authors reported that Cu dissolves into the ionic liquid forming a copper dicyanamide complex and simultaneous deposition of gold on copper takes place. The morphology of the gold deposited on copper had a spherical structure of ~200 nm in size and with increase in temperature, the amount of deposit was found to increase at a faster deposition rate. However, the authors neither studied the adhesive nature of gold on copper nor the speciation of Au^+^ ion or the catalytic applicability of the deposit.

Electroless deposition of platinum nanoparticles was achieved from ionic liquids by a disproportionation reaction (Zhang et al., 2013). Pt was deposited onto glassy carbon from [EMIm]TFSA and [EMIm]BF_4_ ionic liquids containing K_2_[PtCl_4_] and HTFSA or CH_3_SO_3_H. It was observed that without a proton source, no reduction of Pt took place. The anion of the ionic liquid was also shown to affect the formation of Pt nanoparticles wherein it was shown that changing the anion of the ionic liquid from TFSA^−^ to ${\text{BF}}_{4}^{-}$, a small quantity of Pt nanoparticles was obtained. This might be due to a different speciation of Pt in the employed ionic liquids which was not evaluated. Electroless deposition of Pt nanoparticles obtained from [EMIm]TFSA in presence of HTFSA is shown in Figure 3d, which shows Pt nanoparticles of 1.3 nm in diameter. The obtained Pt nanoparticles were used for electrocatalysis of formic acid. Figure 3e shows the electrocatalysis of formic acid to CO_2_ on Pt nanoparticles obtained from [EMIm]TFSA containing HTFSA (solid line in Figure 3e) or CH_3_SO_3_H (dashed line in Figure 3e). It is evident that the oxidation current density of formic acid conversion on Pt nanoparticles deposited from HTFSA and CH_3_SO_3_H is different, which was related to the different facets formed in the nanoparticles. Therefore, it is possible that with proper choice of ionic liquids and reducing agents, high catalytic facet noble metal nanoparticles can be produced by electroless deposition, and exploited in catalysis.

## Electroless Deposition of Non-Noble Metals

Tin (Sn) deposition on Cu by a galvanic displacement reaction was studied in ChCl-H_2_O in the presence of thiourea (Wang et al., 2013b). From the standard electrochemical series, the Cu/Cu^+^ redox potential is more positive compared to the Sn/Sn^2+^, thus the galvanic displacement reaction should not be feasible due to a positive value for change in Gibbs energy. However, in ionic liquids, the speciation of the metal ions can be changed, and the redox potentials can favor the galvanic displacement reaction, thus making the electroless deposition of Sn on Cu feasible. Moreover, it was shown that the rate of deposition of Sn on Cu depends on temperature and concentrations of SnCl_2_ and thiourea in ChCl-H_2_O. On increasing the SnCl_2_ concentration up to 15 g L^−1^ a deposition rate of ~3.2 μm h^−1^ was observed. The addition of thiourea of 90 g L^−1^ marginally improved the rate of deposition to ~3.4 μm h^−1^. The XRD results showed that within 1 min of galvanic displacement at 40°C, prominent peaks of Sn could be distinguished. Sn is a promising anode material for both Li-ion and Na-ion batteries (Liu et al., 2016). However, no attempt was made to use these deposits for battery applications or to understand the mechanism of the galvanic displacement reaction.

Electroless plating of copper on Al and Al-5 wt%Si was shown to be feasible in ChCl-ethylene glycol (ChCl-EG) (Kang et al., 2014, 2015). Although the galvanic displacement reaction of Cu on Al is possible from aqueous solutions, the presence of an oxide layer on Al inhibits the formation of uniform Cu coatings. Furthermore, strong alkaline solutions or F-containing solutions are required to remove the oxide layer from Al to facilitate the electroless deposition process (Ai et al., 2011; Ye et al., 2012). However, in ionic liquids, no F-containing additive was needed. A few Cu(II) salts [CuSO_4_.5H_2_O, CuCl_2_.2H_2_O, and Cu(CH_3_COO)_2_] were tested for the electroless deposition on Al (Kang et al., 2014), among which CuCl_2_.2H_2_O showed the highest solubility in ChCl-EG. Based on UV visible spectroscopy, formation of [CuCl_4_]^2−^ was identified and the galvanic displacement reaction was proposed as shown in Equations (4, 5).

(4) $$\begin{array}{l} Al+3{\left[CuCl_{4}\right]}^{2-}\to \;Al^{3+}+3{\left[CuCl_{2}\right]}^{-}+6Cl^{-} \end{array}$$

(5) $$\begin{array}{l} Al+3{\left[CuCl_{2}\right]}^{-}\to Al^{3+}+3Cu+6Cl^{-} \end{array}$$

Electrochemical noise analysis (ECN) was used to identify the number of stages for the electroless deposition process from the plots of current vs. time from which four stages were identified for the galvanic displacement reaction (Kang et al., 2014). In the first stage the sharp increase in current was attributed to nucleation-growth phenomena followed by a decrease in reaction rate in 2nd stage which led to decrease in current. In third and fourth stages, the current was low but almost constant and was related to galvanic displacement reactions. However, the displacement reaction on Al-5 wt%Si was found to be slightly different (Kang et al., 2015). From the microstructure and elemental map analyses, it was observed that initially Cu displaced only Al and did not form any coating on the silicon regions of the Al-Si alloy. With time, a uniform coating could be obtained both on Al and Al-Si alloy. However, there are a few open questions as to how did Cu(II) displace the Si in the Al-Si alloy which will need further investigation. Furthermore, with change in Si concentration in the Al-Si alloy, it would be interesting to evaluate the electroless deposition process.

To improve the corrosion resistance and mechanical properties of Al, Ni-P coating is usually used from aqueous solutions at temperatures of >80°C. Kang et al. showed that modifying the Al substrate with galvanic displacement of Cu or Zn followed by electroless deposition of Ni-P on the modified substrate led to a better and uniform coating with improved corrosion resistance (Kang et al., 2017). The authors argued that Cu/Zn acted as a catalytic material which improved the deposition of Ni-P. Unfortunately, the microstructural analysis was limited to understanding the surface phenomena without looking into the cross-sectional phenomena of Al-Cu/Zn-Ni-P. It is important to understand the cross-sectional behavior and the grain boundary formed between Al-Cu and Ni-P in order to improve the deposition characteristics. Therefore, further studies are needed to evaluate the complex displacement reaction mechanism.

**Figure 4:**
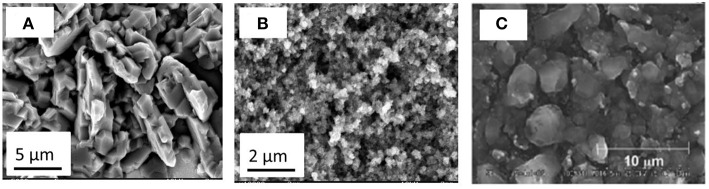
**(A)** Microstructure of electrodeposited Al from [EMIm]Cl/AlCl_3_ at −0.3 V vs. Al and **(B)** from [Py_1,4_]Cl/AlCl_3_ at −0.3 V vs. Al for 1 h **(C)** Al electroless deposited from [EMIm]Cl/AlCl_3_ in presence of LiH as reducing agent. Reproduced from Koura et al. (2008) with permission from The Electrochemical Society.

Al coating on reactive metal surfaces, especially on steel is an attractive technique to prevent corrosion and to improve the conductivity. Al has been electrodeposited on various substrates from ionic liquids (Zein El Abedin et al., 2005; Zein El Abedin and Endres, 2006; Eiden et al., 2009; Giridhar et al., 2012a). AlCl_3_ dissolution in ionic liquids is an exothermic process. Depending on the AlCl_3_ concentration in the ionic liquid, Lewis acid or Lewis basic electrolytes can be formed. Aluminum can be electrodeposited from Lewis acids of 60:40 mol% of AlCl_3_:[EMIm]Cl. The electrodeposits contain micrometer sized Al (Figure 4A).

When changing the electrolyte from AlCl_3_:[EMIm]Cl to AlCl_3_:1-butyl-1-methylpyrrolidinium chloride (AlCl_3_:[Py_1, 4_]Cl) electrodeposition leads to nanocrystalline Al (Figure 4B). The change in the deposit morphology can be attributed to a change in interfacial structure at the EEI (Endres et al., 2010). Similar results have also been observed for the electrodeposition of Al from AlCl_3_ dissolved in ILs containing bis(trifluoromethylsulfonyl)amide (Moustafa et al., 2007) and trifluoromethylsulfonate anions (Giridhar et al., 2012b) with either 1-butyl-1-methylpyrrolidinium or 1-ethyl-3-methylimidazolium cations. By mixing both [EMIm]Cl and [Py_1, 4_]Cl with AlCl_3_, a range of different microstructures could be obtained. Al deposits have been made from various compositions of AlCl_3_:1-ethyl-3-methylimidazolium chloride (AlCl_3_:[EMIm]Cl) and AlCl_3_: 1-butyl-1-methylpyrrolidinium chloride (AlCl_3_:[Py_1, 4_]Cl). The deposits thus obtained possess nanostructures and/or microstructures depending on the amount of 60:40 mol% AlCl_3_:[EMIm]Cl in the mixtures of 60:40 mol% AlCl_3_:[Py_1, 4_]Cl and 60:40 mol% AlCl_3_:[EMIm]Cl. This study clearly showed that the cation of the ionic liquid has a remarkable influence on the particle size of Al electrodeposited from the aforementioned ionic liquids.

Recent studies have shown the possibility of electroless coating of Al using reducing agents in ionic liquids. Koura et al. (2008) used reducing agents such as LiH, LiAlH_4_, or diisobutyl aluminum hydride for the reduction of Al from AlCl_3_-1-ethyl-3-methylimidazolium chloride. The plating time, temperature and concentration of the reducing agent were evaluated for electroless deposition of Al on copper. At 25 and 35°C, a maximum amount of 0.4 mg cm^−2^ of Al was obtained whereas on increasing the temperature to 45°C, the amount decreased to 0.25 mg cm^−2^, which was attributed to the decomposition of the electrolyte. With increase in plating time and concentration of the reducing agent, the authors found that the Al particle size increased, and thicker deposits could be obtained. Among the three reducing agents, the authors found that the addition of diisobutyl aluminum hydride led to a much smoother surface. A comparison of electroless deposited Al (Figure 4C) and electrodeposited Al from the same electrolyte is shown in Figure 4A. It is evident that Al deposits in both cases show particle sizes of ~2–3 μm on the electrode. This shows that electroless deposition in presence of a reducing agent is a simple route to obtain Al on different substrates without the need of additional energy. Furthermore, such a technique might be useful to coat Al on an insulating substrate as well.

A much more detailed study of electroless deposition of Al from AlCl_3_/[EMIm]Cl in presence of diisobutyl aluminum hydride as reducing agent was investigated by Shitanda et al. (2009). The proposed reaction mechanism for the electroless deposition is shown in Equations (6, 7).

(6) $$\begin{array}{l} 4Al_{2}Cl_{7}^{-}+3e^{-}\to 7AlCl_{4}^{-}+Al \end{array}$$

(7) $$\begin{array}{l} 2\left\{{\left(CH_{3}\right)}_{2}-CH-CH_{2}-AlH-CH_{2}-CH-{\left(CH_{3}\right)}_{2}\right\}\to \\ 2\;{\left\{{\left(CH_{3}\right)}_{2}-CH-CH_{2}-Al-CH_{2}-CH-{\left(CH_{3}\right)}_{2}\right\}}^{+}\\ +H_{2}+2\;e^{-} \end{array}$$

The authors analyzed various parameters such as the influence of plating time, temperature, concentration of reducing agent and the concentration of metal ions on the electroless plating, and surface morphology of Al. With increase in plating time at temperatures of 35 and 55°C, the amount of Al deposit increased. However, on performing the experiments at 65°C, the amount of Al decreased due to decomposition of the electrolyte. With time, at lower temperature (35°C) it was shown that the Al crystals grew in size to 2–5 μm and on increasing the temperature to 65°C, a much smoother surface with a smaller grain size (<1 μm) was obtained. The change in AlCl_3_ concentration in the electrolyte also affected the deposit morphology. When changing the mol% of AlCl_3_ from 52 to 58 and 66.7, more Al could be deposited. However, a smoother film could be obtained for 58 mol% AlCl_3_. Finally, with an increase in reducing agent concentration in the electrolyte with a fixed AlCl_3_ concentration, more Al could be plated.

Using the same [EMIm]Cl/AlCl_3_ electrolyte in the presence of diisobutyl aluminum hydride as reducing agent, electroless deposition of Al was also successfully obtained on nickel nanowires (Poges et al., 2018). Using SEM and neutron scattering measurements, the authors showed that a core-shell structure was obtained for electroless deposited Al on Ni nanowires. The Al coating thickness on the nickel nanowires was found to be between 300 and 600 nm. The ferromagnetic magnetization property was found to decrease from 43.3 to 31.4 emu g^−1^ from the magnetic measurement of nickel nanowires and Al coated nickel nanowires, respectively. Although, these studies showed a simple route for Al plating, further investigation on practical systems like steel might be more appropriate.

Al has also been used as a corrosion protection layer for uranium (Egert and Scott, 1987; Jiang et al., 2017). Recently, electroless deposition of Al from [EMIm]Cl/AlCl_3_ on uranium was shown to be an effective method for coating uranium (Jiang et al., 2018). The galvanic displacement reaction of Al on U takes place according to Equation (8).

(8) $$\begin{array}{l} U+4Al_{2}Cl_{7}^{-}\to {Al+U}^{3+}+7AlCl_{4}^{-} \end{array}$$

As uranium dissolves into the electrolyte, simultaneous Al deposition occurs. From UV-visible spectroscopy, the authors evaluated the dissolution rate of U during the displacement reaction. Within the first 20 min of the reaction, the U dissolution rate decreased from 0.03 to 0.02 mol min^−1^ m^−2^. After 120 min of the reaction, an insignificant amount of U was found to dissolve which suggested that most of the U was covered by Al. From a cross-sectional SEM analysis, the authors showed that a 200 nm thick Al deposit was formed on U after 2 h of reaction. Although this study shows a simple route to protect U, the practical application of this process remains arguable due to cleaning procedures required after the deposition process and disposal of the U ions in the solution.

Galvanic displacement of nickel on copper was achieved using a deep eutectic solvent of ChCl and ethylene glycol mixtures (Yang et al., 2016). Nickel was shown to form [NiCl_4_]^2−^ ions in the electrolyte which displaced copper, forming nickel metal and [CuCl_3_]^2−^ ions. It was shown that the displacement reaction takes place in three stages. Initially, copper is stripped resulting in a porous structure along with progressive nucleation of nickel nanoparticles. In the second stage, more copper is dissolved leading to surface cracks along with deposition and growth of nickel. Finally, as the nickel covers the copper surface, the reaction slows down leading to the formation of a porous nickel layer. The obtained nickel was used as a catalytic material for the hydrogen evolution reaction (HER). The HER catalytic activity of nickel was shown to be better than nickel wire and required a lower overpotential of 170 mV vs. RHE for HER. A large exchange current density of 0.186 mA cm^−2^ with a relatively small Tafel slope of 98.5 mV decade^−1^ could be achieved by the obtained porous nickel structures on Cu.

## Electroless Deposition of Alloys and Composite Materials

Alloys and composite materials have been shown to be useful electrode materials for Li-ion and Na-ion batteries as they have lower volume expansion during intercalation/deintercalation processes, high theoretical capacities, and longer life time compared to carbon electrodes and to their metal counterparts (Zhang, 2011; Obrovac and Chevrier, 2014; Lahiri and Endres, 2017). Alloys and composites are in general deposited using high temperature techniques, vacuum techniques, sol-gel processes, or by electrodeposition (Zhang, 2011; Obrovac and Chevrier, 2014; Lahiri and Endres, 2017). Electroless deposition has also been used to develop alloys and composites.

A copper antimonide (Cu-Sb) alloy has been used as anode for both Li and Na-ion batteries (Lahiri and Endres, 2017). The Cu_2_Sb alloy has been prepared by galvanic displacement reaction from ionic liquids (Lahiri et al., 2018b). It was observed that on exposing a copper substrate to SbCl_3_-[EMIm]TFSA, Cu_2_Sb nanoplates formed instantaneously. From mass spectroscopic analysis, it was shown that SbCl_3_ in the electrolyte forms a [SbCl_2_(TFSA)_3_]^2−^ complex which had a lower reduction potential compared to Cu/Cu^+^ in the same electrolyte and triggered the electroless deposition to take place. The electroless deposition process was studied using *in situ* X-ray photoelectron spectroscopy (XPS), X-ray diffraction (XRD), and *in situ* atomic force microscopy (AFM). From XPS and XRD measurements, formation of CuCl was observed which suggested that a three-electron transfer process was taking place for the formation of Cu_2_Sb. From *in situ* AFM, the deposition rate was estimated to be between 0.4 and 1.65 nm min^−1^. The electroless deposited Cu_2_Sb nanoplates were also tested for Li and Na ion batteries with an ionic liquid electrolyte which showed a promising battery performance. However, the question remains as to why Cu_2_Sb formed a nanoplate structure by electroless deposition. Further studies using transmission electron microscopy along with selected area electron diffraction (SAED) might reveal the crystal growth mechanism.

It was interesting that Fe could also be deposited on Cu by galvanic displacement reaction from 0.15 M FeCl_2_ dissolved in a mixture of 1-butylpyrrolidine and AlCl_3_ (1:1.2 mol ratio) containing toluene at 60°C (Lahiri et al., 2018b). Although from aqueous standard electrochemical series, such a galvanic displacement reaction is impossible, these reactions become feasible in ionic liquids due to the difference in speciation. The electroless deposited Fe was confirmed with XRD measurements. Further studies regarding the influence of FeCl_2_ concentration in the eutectic-based ionic liquid is required to understand the reaction mechanism of the galvanic displacement reaction process. Furthermore, such studies will be useful to improve the deposition as well as to develop different nanostructures.

**Figure 5:**
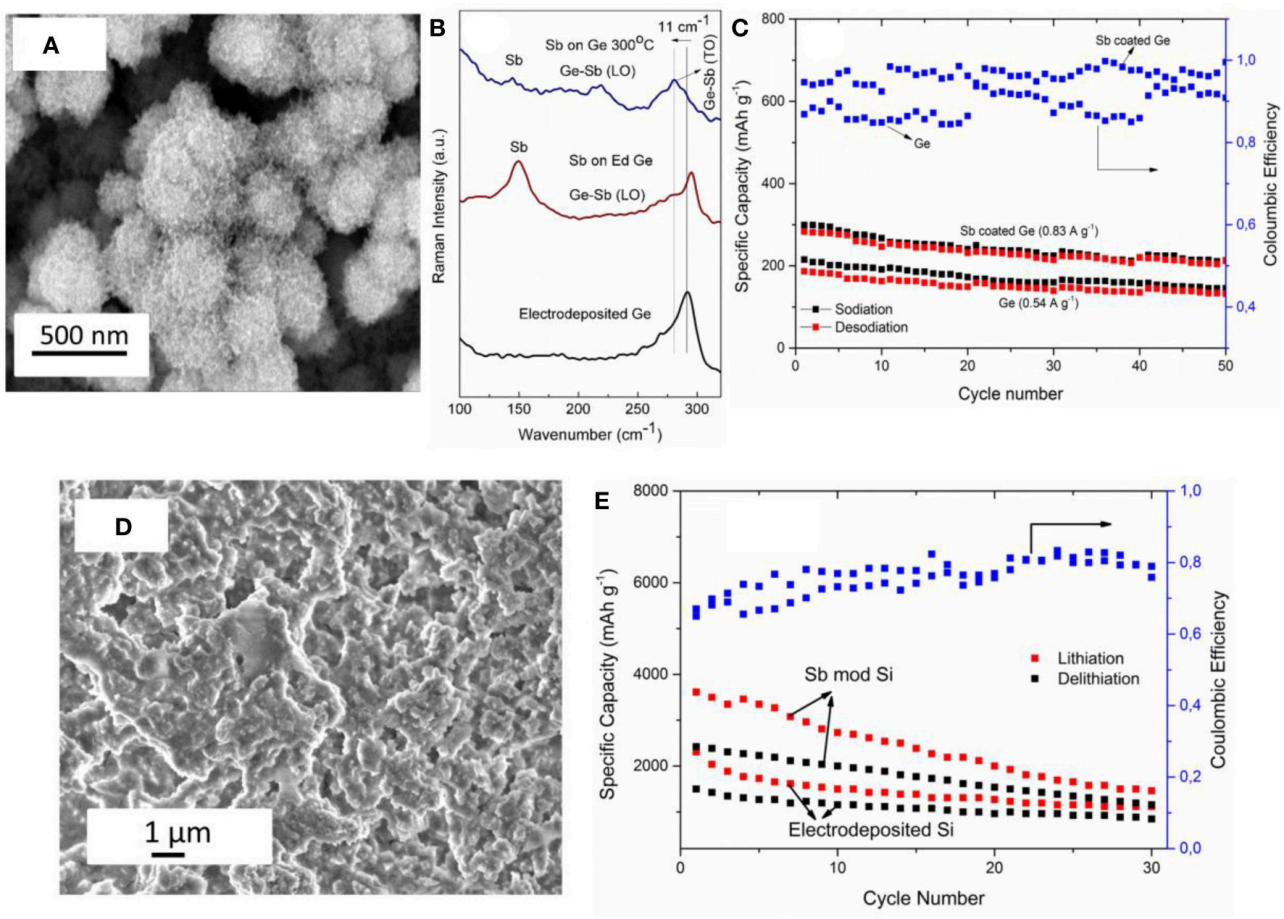
**(A)** Microstructure of Sb modified Ge nanoparticles **(B)** Raman spectra of electrodeposited Ge (black line), electroless deposited Sb on Ge (red line) and after annealing to 300°C (blue line) **(C)** SEM of electroless deposition Sb on silicon **(D)** Charge-Discharge cycles of electrodeposited Ge and Sb modified Ge for Na-ion batteries in 1 M NaFSA-[Py_1,4_]FSA **(E)** Charge-Discharge cycles of electrodeposited Si and Sb modified Si for Li-ion batteries in 1 M LiTFSA-[Py_1,4_]TFSA. Reproduced from Lahiri et al. (2016) and Lahiri et al. (2017) with permission from The Royal Society of Chemistry and American Chemical Society. Copyright 2017 American Chemical Society.

Unlike Cu_2_Sb, interestingly, semiconducting amorphous gallium antimonide (GaSb) was obtained by electroless deposition of Sb on Ga from ionic liquids (Lahiri et al., 2015b). Galvanic displacement of Sb on Ga was performed from two different ionic liquids with [Py_1, 4_]TFSA and [EMIm]TFSA. The change in the cation of the ionic liquid influenced the morphology of GaSb deposit. The bandgap of the GaSb deposit was found to be ~0.9 eV. From *in situ* AFM studies, the deposition rate was reported to be 2.3 nm min^−1^. Furthermore, formation of GaSb nanowires was shown to be possible using electroless deposition. As the open circuit potential of Sb/Sb^3+^ is more positive compared to Si and Ge in the same ionic liquid, Sb modification of both electrodeposited Si and Ge was achieved (Lahiri et al., 2016, 2017). The electroless deposition of Sb on Ge from 0.1 M SbCl_3_-[Py_1, 4_]TFSA led to the formation of Sb nanoparticles. Figure 5A shows the microstructure of the deposit wherein Ge nanoparticles of 200–300 nm are covered with Sb nanoparticles of < 50 nm in size. XPS and Raman studies showed the formation of Ge_x_Sb_1−x_. From Raman spectroscopy (Figure 5B) at room temperature, both Ge and Sb peaks are observed with a shoulder showing the formation of Ge_x_Sb_1−x_. With annealing, a clear shift in the Ge peak is seen confirming the formation of Ge_x_Sb_1−x_. As both Ge and Sb are possible anode materials for Na-ion batteries, Ge and Sb modified Ge were tested as battery electrodes with an ionic liquid electrolyte (Lahiri et al., 2016). Figure 5C compares 50 charge-discharge cycles of electrodeposited Ge and Sb modified Ge with sodium bis(fluorosulfonyl)amide (NaFSA) in [Py_1, 4_]FSA ionic liquid as electrolyte. It is evident that modifying the Ge with Sb led to a higher Na capacity storage even at higher current densities of 0.83 A g^−1^ compared to Ge which was cycled at a current density of 0.54 A g^−1^. Similar to the modification of Ge with Sb, electrodeposited silicon was also modified by Ag and Sb as it was found that the open circuit potentials of both Ag/Ag^+^ and Sb/Sb^3+^ are more positive to Si(IV)/Si in the ionic liquid (Lahiri et al., 2017). From quartz crystal microbalance (QCM) measurements, the rate of deposition for Sb on Si was found to be 2.7 ng sec^−1^ for the first 1,000 s which then decreases to 0.4 ng sec^−1^. In comparison, the deposition of Ag on Si was 6.2 ng sec^−1^ which decreased to 1.8 ng sec^−1^ after 1,000 s. Although a three times increase in the deposition of Ag is expected compared to Sb deposition, it was shown that due to difference in speciation of Ag and Sb salts in an ionic liquid, the deposition rates differed. In comparison to the formation of Sb nanoparticles in the case of Ge (Figure 5A), a layered structure of Sb has been seen for Si (Figure 5D). This suggests that the nucleation-growth process of Sb on the two semiconductors is different. The electrodeposited Si and Sb modified Si was also tested as an anode for Li-ion battery.

Figure 5E shows the charge-discharge curves of the electrodeposit in 1 M LiTFSA-[Py_1, 4_]TFSA cycled at 0.25 C. The Sb modified Si showed a capacity of over 2,000 mAh g^−1^ for the first 10 cycles which decreased to 1,100 mAh g^−1^ at the 30th cycle. Compared to the modified Si, the electrodeposited silicon showed a lower Li storage capacity. Based on the results of Sb electroless deposition, further investigations are needed regarding long term cycling stability of the anode material. Furthermore, during battery testing, the lithiation/delithiation processes need to be evaluated in order to understand how the alloying of Sb/semiconductor changes.

## Challenges and Limitations of Ionic Liquids for Electroless Deposition

The electroless deposition process in ionic liquids has been shown to be an autocatalytic deposition process which occurs at the metal/electrolyte interface by the reduction of metallic ions/complexes in the presence or in the absence of externally added reducing agents. However, this process suffers from a few limitations such as slow ion transport/diffusion of metal ion species/complexes and slow kinetics of the deposition process at room temperature. Furthermore, issues regarding adhesion of the film, surface selectivity, control over deposit morphology, and purity of the films exist. Therefore, future efforts need to be directed toward overcoming these limitations in order to develop a successful method for material synthesis using this simple energy-free methodology.

For example, in the galvanic displacement reaction the substrate serves as a reducing agent and an electron source for the reduction of metal ion species/complexes. Here, the rate of the deposition depends on the transport of the ions to the interface and also on the rate of dissolution of the substrate. Although an increase in temperature does improve the reaction kinetics and the diffusion processes, however, adhesion of the reduced metal on the substrate might not always be sufficiently good. Therefore, an optimum condition between reaction kinetics, diffusion parameters, temperature, and adhesion of the films need to be balanced.

For better adhesion as well as to develop different nanostructures, surface selectivity plays a key role during the galvanic displacement process. A proper surface modification could lead to a good metal finish. Furthermore, it could promote development of interesting nanostructures which can then be applied in batteries and catalysis. The crystal plane of the surface will also influence the galvanic displacement reaction and deposit morphology which has not yet been studied in ionic liquids.

The grain size and growth of the deposited films for galvanic displacement reactions depend on different factors such as immersion time and temperature, rate of deposition, activation sites on the substrate and speciation in ionic liquids. Control over these factors in electroless deposition is challenging. Usually, large grains or thick films have been obtained at longer immersion times and at higher temperatures. However, by controlling the reaction kinetics and by addition of surfactants/mixture of ionic liquids, it could be possible to spatially control the grains/films which would be of interest for catalytic applications. Furthermore, it might be possible to develop various nanostructures which can then be applied as electrodes in batteries and catalysis/electrocatalysis.

One major issue with electroless deposition from ionic liquids is the cleaning process to obtain the deposited film without any contaminants. Usually, salts of the metallic substrate and trapped ionic liquid are present in the deposits which are difficult to remove. Suitable solvents are required to remove these salts/ionic liquids. It is of interest to design tuneable ionic liquids which can be used to dissolve the metal salts formed during galvanic displacement reaction and the ionic liquid itself being soluble in simple organic solvents.

Finally, the ultimate challenge is to develop an electrochemical series for ionic liquids. This would require a universal reference electrode. At present a universal reference electrode for ionic liquids does not yet exist. Depending on the individual experiment a Pt quasi-reference electrode is an acceptable compromise.

## Summary and Future Perspectives

In the last decade, electroless deposition from ionic liquids has been identified as a promising route for developing various metal and semiconductor nanostructures, some of which have shown to be useful in catalysis and as electrodes for batteries. Compared to aqueous solutions, the electroless deposition of noble metals from ionic liquids does not stop after a few monolayers and leads to deposits with thicknesses between 100 nm and 10 μm. Furthermore, as the speciation of metals can be modified in ionic liquids, the electroless deposition does not always follow the standard electrochemical series. Various parameters such as the metal precursors, the employed ionic liquid, temperature, and concentration were shown to affect the deposit morphology, particle size as well as the deposition rate. The presence of an ionic liquid also affected the crystallographic planes. This was exemplified in the electroless deposition of Pt nanoparticles from different ionic liquids wherein it was shown that the formic acid oxidation current differed and depended on the low index planes of the Pt nanoparticles. Similarly, it was shown that depending on the Au precursor and the ionic liquid, triangular gold nanoplates could be formed. Therefore, ionic liquids can be used as a medium to control the crystallographic facets, which turn out to be a useful approach to improve catalytic reactions. In the case of electroless plating of noble metals (e.g., Ag) for PCBs, the adhesion of these metals is an important factor for both Au and Ag deposition. The adhesion properties of the electroless coated metals have been shown to be dependent on the metal ion speciation and the ionic liquid played a vital role to obtain a good metal finish.

Besides noble metals, the possibility of coating non-noble metals especially aluminum on steel, which is an industrially important technique for corrosion prevention makes electroless deposition a lucrative technique to be exploited. For electrodeposition, it was shown that by changing the cation of the ionic liquid, the morphology and the grain size of the deposit could be altered which will in-turn influence the corrosion properties of the coating. Such simple modifications become a useful tool to obtain better corrosion prevention materials which are yet to be investigated by an electroless deposition route from ionic liquids.

Electroless deposition in ionic liquids was also utilized for developing intermetallics and composites which have potential applications as electrode materials for batteries. Various alloy nanostructures have been obtained by electroless deposition from ionic liquids and were shown to have good Li and Na storage properties. The ease of the process for materials synthesis makes it a beneficial technique for developing other alloy nanostructures which can be tested as electrodes for batteries.

Thus, based on the above literature, it is clear that some limitations and challenges remain in the field of electroless deposition from ionic liquids. However, it is evident that electroless deposition in ionic liquids has tremendous potential for developing functional materials both for catalysis and for batteries which are yet to be exploited. New domains comprising of mixtures of ionic liquids, addition of organic solvents and water in ionic liquids for electroless deposition of metals and semiconductors are yet to be investigated which might lead to a facile synthesis technique to obtain novel nanostructures. The possibility of developing porous materials and one-dimensional nanostructures by template-assisted techniques would also bring new perspectives to electroless deposition from ionic liquids. Finally, the challenge will be to develop ionic liquid-based electrolytes with smart functionalities that could be used to develop specific functional materials.

## Author Contributions

All authors listed have made a substantial, direct and intellectual contribution to the work, and approved it for publication.

### Conflict of Interest Statement

The authors declare that the research was conducted in the absence of any commercial or financial relationships that could be construed as a potential conflict of interest.
